# Supplementing narasin or monensin to control coccidiosis in naturally infected calves

**DOI:** 10.1093/tas/txae069

**Published:** 2024-04-26

**Authors:** Tiago Leiva, Reinaldo F Cooke, Pedro V F Lasmar, Rodrigo L Valarelli, José M C De Simas, Dina Maria B Zapa, Luiz Felipe M Couto, Luciana M Heller, Welber D Z Lopes

**Affiliations:** Elanco Animal Health, Sao Paulo, SP 04703-002, Brazil; Department of Animal Science, Texas A&M University, College Station, TX 77845USA; Elanco Animal Health, Sao Paulo, SP 04703-002, Brazil; Elanco Animal Health, Sao Paulo, SP 04703-002, Brazil; Elanco Animal Health, Sao Paulo, SP 04703-002, Brazil; Instituto de Patologia Tropical e Saúde Publica, Universidade Federal de Goiás, Goiânia, GO 74605-050, Brazil; Instituto de Patologia Tropical e Saúde Publica, Universidade Federal de Goiás, Goiânia, GO 74605-050, Brazil; Instituto de Patologia Tropical e Saúde Publica, Universidade Federal de Goiás, Goiânia, GO 74605-050, Brazil; Instituto de Patologia Tropical e Saúde Publica, Universidade Federal de Goiás, Goiânia, GO 74605-050, Brazil

**Keywords:** calves, coccidiosis, ionophores, monensin, narasin

## Abstract

This experiment compared narasin and monensin as anticoccidials for calves naturally infected with *Eimeria* spp. Twenty-four weaned, non-castrated male calves (*Bos indicus* × *B. taurus* cross) were assigned to this experiment (days −8 to 42). All calves were infected by *Eimeria* spp. according to oocyst count per gram (**OPG**) from fecal samples collected on days −8 and −7 (average 1,059 ± 101 oocysts/g). Calves were housed in individual pens, received corn silage, mineral mix, and water for ad libitum consumption, in addition to a grain-based supplement at 200 g/head daily. Fecal samples were collected on days −2 and −1 for OPG, and results averaged as initial OPG value. Calves were blocked according to initial OPG into eight blocks of three calves each, ranked within each block according to body weight (**BW**) recorded on day −1, and assigned to receive narasin (**NAR;** 0.8 mg/kg of BW), monensin (**MON;** 1 mg/kg of BW), or no ionophore (**CON**; negative control). Ionophores were added to the grain-based supplement, and offered from days 0 to 42 of the experiment. Calf BW was recorded on days 7, 14, 21, 28, 35, and 42. Fecal samples were collected on days 6 and 7, 13 and 14, 20 and 21, 26 and 27, 34 and 35, and 41 and 42 for OPG analysis, and results from samples collected on consecutive days were averaged. Aliquoted fecal samples were also pooled across calves from the same treatment and collection days, and used to determine the prevalence of individual species of *Eimeria*. No treatment effects were detected (*P *≥ 0.51) for calf BW or growth rate. A treatment × day interaction was detected (*P *< 0.01) for OPG, as NAR and MON calves had less (*P *< 0.01) OPG compared with CON calves beginning on day 7. The OPG was also less (*P *≤ 0.03) in MON compared with NAR calves on days 7, 14, and 28, but did not differ (*P *≥ 0.48) on days 21, 35, and 42. The anticoccidial efficacy of NAR and MON did not differ (*P *≥ 0.16) when calculated across all *Eimeria* spp., or according to prevalence of *E. bovis* and *E. alabamensins*. A treatment × day interaction was detected (*P *= 0.04) for anticoccidial efficacy to *E. alabamensis*, which was greater (*P *< 0.01) in MON calves on days 7 and 14 and did not differ (*P *≥ 0.40) afterward. Collectively, both ionophores were similarly effective in controlling coccidiosis upon completion of the 42-d study, although the anticoccidial effects of monensin were noted earlier in the experiment. Nonetheless, these results corroborate narasin as an efficient anticoccidial ionophore for naturally infected calves.

## Teaser

Coccidiosis is an infectious disease caused primarily by the *Eimeria* protozoa, leading to considerable morbidity and mortality in young cattle. This study evaluated the use of ionophores (monensin and narasin) to control coccidiosis in calves naturally infected with *Eimeria.*

## Introduction

Bovine coccidiosis is an infectious disease caused primarily by protozoa of the genus *Eimeria*, leading to considerable morbidity and mortality in young cattle (Noack et al., 2019; Bangoura and Bardsley, 2020). *Eimeria* are unicellular, host-specific, and intracellular parasites that are transmitted by fecal-oral route, when ingested sporulated oocysts invade the intestinal epithelium (Lassen and Ostergaard, 2012). Within the several *Eimeria* spp. identified in cattle (Cruvinel et al., 2018), *E. bovis*, *E. zuernii,* and *E. alabamensis* are considered pathogenic and causative of the disease (Daugschies and Najdrowski, 2005).

The severity of coccidiosis depends on many factors including species of *Eimeria*, oocyst load, host age, and immunocompetence (Cornelissen et al., 1995). Clinical coccidiosis causes direct economic losses from morbidity and mortality, whereas the subclinical disease impairs growth performance and productivity of young cattle (Fitzgerald, 1980; Cornelissen et al., 1995). Therefore, several methods to prevent and control coccidiosis are adopted in cattle operations, particularly dietary inclusion of ionophores (Noack et al., 2019). These feed additives are commonly used in cattle diets to improve feed efficiency, but are also known to neutralize *Eimeria* spp. by disrupting ion transport across its cell membrane (Noack et al., 2019).

Monensin is a monovalent ionophore widely used to enhance growth performance while controlling coccidiosis in cattle (Vendramini et al., 2018; Oliveira et al., 2020). Narasin is another monovalent ionophore that improves cattle productivity (Marques and Cooke, 2021; Miranda et al., 2021; Miszura et al., 2023), although its anticoccidial effects still warrant investigation. Narasin is known to effectively mitigate coccidiosis in poultry (Chapman and Rathinam, 2022); hence, we hypothesized that this ionophore can also be used as an anticoccidial for cattle. To test this hypothesis, this experiment evaluated the effects of narasin in controlling coccidiosis in calves naturally infected with *Eimeria* spp.

## Materials and Methods

All animals utilized in this experiment were cared for in accordance with acceptable practices and experimental protocols reviewed and approved by the Universidade Federal de Goiás, Comitê de Ética no Uso de Animais (#CEUA/UFG—051/20).

### Animal Management and Dietary Treatments

Twenty-four weaned, non-castrated male calves (½ Nelore [*Bos indicus*] × ½ Girolando [*B. indicus*-influenced] cross) were assigned to this experiment (days −8 to 42). Calf body weight (**BW**) and age on day −8 averaged, respectively, 97.6 ± 6 kg and 6.1 ± 0.6 mo. Calves did not receive any anticoccidial treatments nor dietary ionophores from birth to the beginning of the experiment. All calves were individually diagnosed as naturally infected by *Eimeria* spp., according to oocyst count per gram (**OPG**; Ueno and Gonçalves, 1988) from fecal samples collected on days −8 and −7 (average 1,059 ± 101 oocysts/g; minimum value of 526 oocysts/g). A threshold of 500 oocysts/g of pathogenic *Eimeria* spp. (*E. bovis*, *E. zuernii*) is used to classify coccidiosis as a health concern in cattle herds (Bangoura and Bardsley, 2020). Calves were housed in individual pens (9 m^2^) with concrete floors bedded with shaved wood, and received corn silage, commercial mineral mix, and water for ad libitum consumption throughout the experiment. Calves also received a limit-fed supplement (60% corn, 39.5% soybean meal, 0.5% urea; as-fed basis) at 200 g/head daily.

Calves were sampled for feces again on days −2 and −1 for OPG (Ueno and Gonçalves, 1988), and results averaged as initial OPG value. Calf BW was recorded on day −1, which was considered initial BW. Calves were blocked according to initial OPG into eight blocks of three calves each, ranked within each block according to initial BW, and then assigned to receive narasin (**NAR**), monensin (**MON;** positive control), or no ionophore (**CON**; negative control) in a manner that treatment groups had equivalent initial OPG and BW. Narasin and monensin were added to their respective supplements at, respectively, 0.8 mg/kg of BW (6.7 mg of Zimprova^TM^/kg of BW daily; Elanco Saúde Animal; São Paulo, Brazil) and 1 mg/kg of BW (5 mg of Rumensin^TM^/kg of BW daily; Elanco Saúde Animal). Treatments were offered from days 0 to 42 of the experiment.

### Sampling

Samples of all feed ingredients were every 2 wk, pooled across weeks, and analyzed for nutrient content by a commercial laboratory (3R labs; Lavras, Minas Gerais, Brasil). Samples were analyzed by wet chemistry procedures for concentrations of CP (method 984.13; AOAC, 2006), ADF (method 973.18 modified for use in an Ankom 200 fiber analyzer, Ankom Technology Corp., Fairport, NY; AOAC, 2006), and NDF (Van Soest et al., 1991; modified for Ankom 200 fiber analyzer, Ankom Technology Corp.). The nutritive value of the corn silage was (dry matter basis) 1.37 Mcal/kg of net energy for maintenance, 0.90 Mcal/kg of net energy for growth, and 7.9% of crude protein. The nutritive value of the supplement was (dry matter basis) 2.22 Mcal/kg of net energy for maintenance, 1.46 Mcal/kg of net energy for growth, and 27.1% of crude protein. The mineral mix contained (dry matter basis) 22% Ca, 7.5% P, 6.5% Na, 3.6% Mg, 0.115% Cu, 0.400% Zn, 0.003% Co, 1.0% K, 0.220% Mn, and 0.003% Se.

Calf BW was recorded again on days 7, 14, 21, 28, 35, and 42 of the experiment, and these values were used to adjust the inclusion of NAR and MON into the supplements on a weekly basis. Growth rate of each calf was modeled by linear regression of BW against sampling days (days −1 to d 42), and each regression coefficient was used as individual growth response. Fecal samples were collected again on days 6 and 7, 13 and 14, 20 and 21, 26 and 27, 34 and 35, and 41 and 42 of the experiment from all calves. Fecal samples were obtained directly from the rectum, stored on ice, and maintained at 4 °C until processing (within 48 h of collection). An aliquot of each sample was analyzed for OPG according to the study by Ueno and Gonçalves (1988). The OPG results from samples collected on consecutive days were averaged to minimize variability, and averaged values are referred to as days −1, 7, 14, 21, 28, 35, and 42 (as BW assessment) to facilitate description of results. The anticoccidial efficacy of treatments was determined according to the OPG of calves within each block and collection day as: Efficacy (%) = (OPG of CON—OPG of NAR or MON)/ OPG of CON (Lopes et al., 2014).

Another aliquot of each fecal sample (5 g each) was pooled across calves from the same treatment and collection days, and used for determination of *Eimeria* spp. prevalence (Ueno and Gonçalves, 1988). Briefly, pooled fecal samples were filtrated using a folded cheesecloth, the filtrate was centrifuged at 250 *g* for 10 min, and the supernatant was added to a petri dish containing 2.5% potassium dichromate. After 10 d, oocysts were recovered upon centrifugation at 250 *g* for 10 min in a saturated sugar solution, and washed with distilled water. The *Eimeria* spp. identification was performed using a light microscopy coupled to a computerized system (Leica Microsystems; Wetzlar, Germany) through the evaluation of shape, color, presence, or absence of micropyle and micropylar hood, oocysts size, and morphological sporocysts characteristics (Duszynski and Wilber 1997; Daugschies and Najdrowski, 2005). The anticoccidial efficacy according to individual *Eimeria* species was calculated based on the equation previously described (Lopes et al., 2014), and the prevalence of each species identified (Zapa et al., 2022).

### Statistical Analysis

All data were analyzed with calf as the experimental unit, and calf(treatment) as random variable. Quantitative data were analyzed using the MIXED procedure of SAS (SAS Inst. Inc., Cary, NC), and binary data were analyzed using the GLIMMIX procedure of SAS (SAS Inst. Inc.). The OPG of each calf (observed egg count + 1) was log-transformed before statistical analyses and reported as log10 (Oliveira et al., 2020). All model statements included the effects of treatment, in addition to day and the treatment × day interaction for repeated measures. The specified term used in the repeated statements was day, the subject was calf(treatment), and the covariance structure used was autoregressive, which provided the best fit for these analyses according to the lowest Akaike information criterion. Results are reported as least square means, and were separated using PDIFF. Significance was set at *P* ≤ 0.05 and tendencies were determined if *P* > 0.05 and ≤ 0.10.

## Results and Discussion

**Figure 1.**
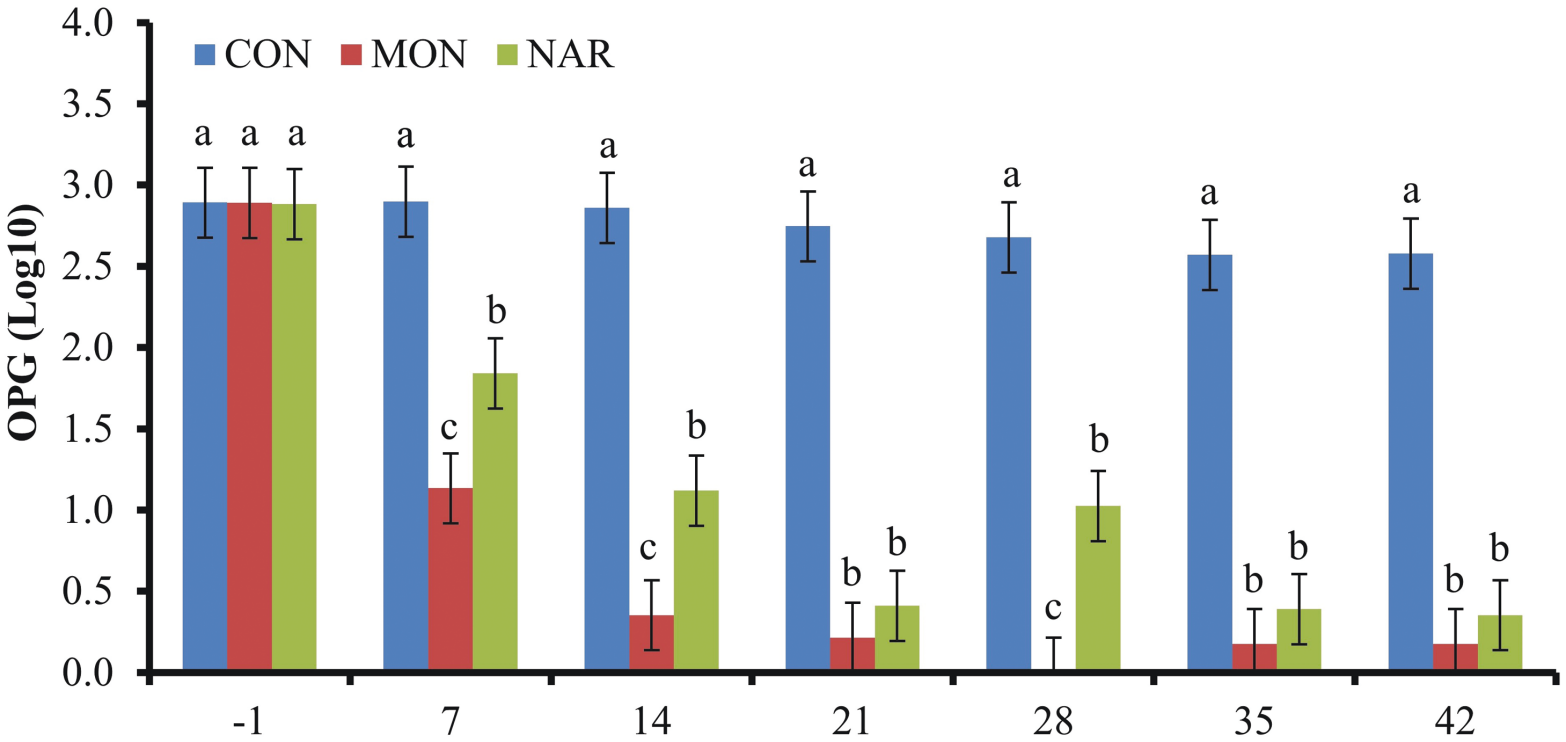
Oocysts of *Eimeria spp.* per gram of feces (**OPG**; Ueno and Gonçalves, 1988) in naturally infected calves receiving narasin (**NAR**; *n* = 8), monensin (**MON**; *n* = 8), or no ionophore (**CON**; *n* = 8). Treatments were individually offered to calves from days 0 to 42 of the experiment via a limit-fed (200 g/head daily) grain-based supplement. Fecal samples were collected on consecutive days (days −2 and −1, 6 and 7, 13 and 14, 20 and 21, 27 and 28, 34 and 35, and 41 and 42). The OPG from samples collected in consecutive days (observed egg count + 1) were averaged, log-transformed (Oliveira et al., 2020), and referred as days −1, 7, 14, 21, 28, 35, and 42 to facilitate description of results. A treatment × day interaction was detected (*P *< 0.01). Within days, values with different letters (a,b,c) differ at *P *≤ 0.02.

Calves across all treatments were naturally infected with *Eimeria* spp. based on initial fecal samples (Table 1), and initial OPG values did not differ (*P *≥ 0.97) among CON, MON, and NAR calves as designed (Figure 1). Initial fecal samples also indicate that calves were primarily infected with *E. bovis* and *E. zuernii* (Table 2), which are the most pathogenic species of the *Eimeria* genus (Daugschies and Najdrowski, 2005). Hence, this experiment successfully tested the research hypothesis and objectives in a population that represents young cattle experiencing coccidiosis.

**Table 1.** Performance responses and fecal presence of *Eimeria spp.* oocysts in naturally infected calves receiving narasin (**NAR**; *n* = 8), monensin (**MON**; *n* = 8), or no ionophore (**CON**; *n* = 8).^*^

Item	CON	MON	NAR	SEM	*P-value*
BW parameters^†^					
Initial BW (day 0), kg	93.5	105.2	95.7	10.0	0.68
Final BW (day 42), kg	102	108	100	11.0	0.87
Growth rate, kg/d	0.262	0.127	0.218	0.087	0.54
Samples with presence of *Eimeria spp.* oocysts^‡^					
Initial (day −1), %	100	100	100	11.6	1.00
day 7, %	100^a^	62.5^b^	75.0^ab^	11.6	0.07
day 14, %	100^a^	25.0^b^	50.0^b^	11.6	<0.01
day 21, %	100^a^	12.5^b^	25.0^b^	11.6	<0.01
day 28, %	100^a^	0.0^c^	62.5^b^	11.6	<0.01
day 35, %	100^a^	12.5^b^	25.0^b^	11.6	<0.01
Final (day 42), %	100^a^	12.5^b^	25.0^b^	11.6	<0.01

^*^Treatments were individually offered to calves from days 0 to 42 of the experiment via a limit-fed (200 g/head daily) grain-based supplement. Narasin and monensin were offered at, respectively, 0.8 mg/kg of BW (6.7 mg of Zimprova^TM^/kg of BW daily; Elanco Saúde Animal; São Paulo, Brazil) and 1 mg/kg of BW (5 mg of Rumensin^TM^/kg of BW daily; Elanco Saúde Animal). A treatment × day interaction was detected (*P *< 0.01) for presence of *Eimeria spp*. oocysts in fecal samples but not (*P ≥ *0.55) for BW. Treatment means with different superscripts (a,b) differ at *P *≤ 0.05.

^†^Body weight (**BW**) was unshrunk and recorded weekly during the experiment. Individual growth rate was modeled by linear regression of BW against sampling days (days 0 to d 42).

^‡^Fecal samples were collected on consecutive days (days −2 and −1, 6 and 7, 13 and 14, 20 and 21, 27 and 28, 34 and 35, and 41 and 42) and analyzed for *Eimeria spp.* oocysts per gram of feces (**OPG**; Ueno and Gonçalves, 1988). Values of OPG from samples collected in consecutive days were averaged, and referred as days −1, 7, 14, 21, 28, 35, and 42 (as BW assessment) to facilitate description of results.

**Table 2.** Prevalence (as %) of *Eimeria spp*. in fecal samples from naturally infected calves receiving narasin (**NAR**; *n* = 8), monensin (**MON**; *n* = 8), or no ionophore (**CON**; *n* = 8).^*^

Item	*E. bovis*	*E. zuernii*	*E. alabamensis*	*E. ellipsoidalis*	*E. cylindrica*	*E. subspherica*
CON						
Initial	59.5	16.8	3.00	12.3	7.66	0.67
day 7	63.7	18.3	2.33	12.0	3.67	0.00
day 14	57.7	20.0	4.33	7.33	5.67	5.00
day 21	73.0	16.0	3.67	5.33	2.00	0.00
day 28	68.7	16.7	5.00	9.67	0.00	0.00
day 35	65.7	15.3	3.33	4.33	4.67	6.67
day 42	72.0	12.7	7.67	4.00	2.67	1.00
MON						
Initial	59.5	16.8	3.00	12.3	7.66	0.67
day 7	44.2	6.56	11.5	31.1	6.56	0.00
day 14	22.0	8.26	13.7	24.8	22.9	8.26
day 21	54.2	4.93	26.7	9.86	0.00	4.23
day 28	0.00	0.00	0.00	0.00	0.00	0.00
day 35	0.00	0.00	0.00	0.00	0.00	0.00
day 42	29.9	24.8	0.00	7.69	24.8	12.8
NAR						
Initial	59.5	16.8	3.00	12.3	7.66	0.67
day 7	52.1	12.7	11.3	21.1	2.82	0.00
day 14	53.2	13.8	13.8	19.1	0.00	0.00
day 21	42.8	7.14	3.57	32.1	14.3	0.00
day 28	16.7	11.4	12.3	35.1	24.5	0.00
day 35	34.7	0.00	18.9	27.4	18.9	0.00
day 42	41.2	17.6	0.00	23.5	17.6	0.00

^*^Fecal samples were collected on consecutive days (days −2 and −1, 6 and 7, 13 and 14, 20 and 21, 27 and 28, 34 and 35, and 41 and 42) of the experiment. Results from samples collected in consecutive days were averaged, and referred as days −1, 7, 14, 21, 28, 35, and 42 to facilitate description of results. Samples were pooled across calves from the same treatment and collections days, and used for determination of *Eimeria* spp. prevalence (Ueno and Gonçalves, 1988).

Treatment × day interactions were detected (*P *< 0.01) for OPG (Figure 1) and proportion of calves diagnosed with *Eimeria* spp. oocysts (Table 1). Calves receiving NAR and MON had less (*P *< 0.01) OPG compared with CON calves after initiation of treatment administration. Proportion of samples with *Eimeria* spp. oocysts were greater (*P *≤ 0.02) for CON compared with MON and NAR calves beginning on days 7 and 14, respectively. Calves receiving MON also had less (*P *≤ 0.03) OPG on days 7, 14, and 28, as well as less proportion of samples with *Eimeria spp.* oocysts on day 28 compared with NAR calves. Nonetheless, OPG and proportion of samples with *Eimeria spp.* oocysts at the end of the experiment did not differ (*P *≥ 0.44) between MON and NAR calves, and were less for both treatments compared with CON (*P *< 0.01). These results indicate that MON and NAR successfully controlled coccidiosis in naturally infected calves upon a 42-d feeding period, although the anticoccidial effects of MON were noted earlier compared with NAR. Monensin was approved in the United States in 1990 for prevention and control of coccidiosis in cattle (FDA, 2004), and such effects are still reported in recent research (Vendramini et al., 2018; Oliveira et al., 2020, 2021). Narasin was approved in 1986 for coccidiosis control in poultry (Anadón and Martínez-Larrañaga, 2014), but has not been evaluated nor approved as anticoccidial for cattle. Monensin and narasin influence the initial phase of the *Eimeria* life cycle, being effective against sporozoites and merozoites in the motile stages. Both ionophores hinder the development of sporozoites by increasing intracellular concentrations of Na^+^ in their cell membranes, and cause the cell membrane of merozoites to burst by promoting activity of Na^+^/K^+^/ATPase (Noack et al., 2019). Several antimicrobials and alternatives to medication have been developed to control coccidiosis in cattle (Bangoura and Bardsley, 2020). However, ionophores remain widely used due to the slow development of resistance, prevention of clinical disease while cattle acquire natural immunity to *Eimeria* spp., and the established benefits to ruminal fermentation and feed efficiency (Chapman et al., 2010; Marques and Cooke, 2021). Ionophores cause biophysical changes in the membrane of *Eimeria* spp., which would require major adaptations in their membrane structure via multiple gene mutations for resistance to develop (Chapman and Rathinam, 2022).

**Figure 2.**
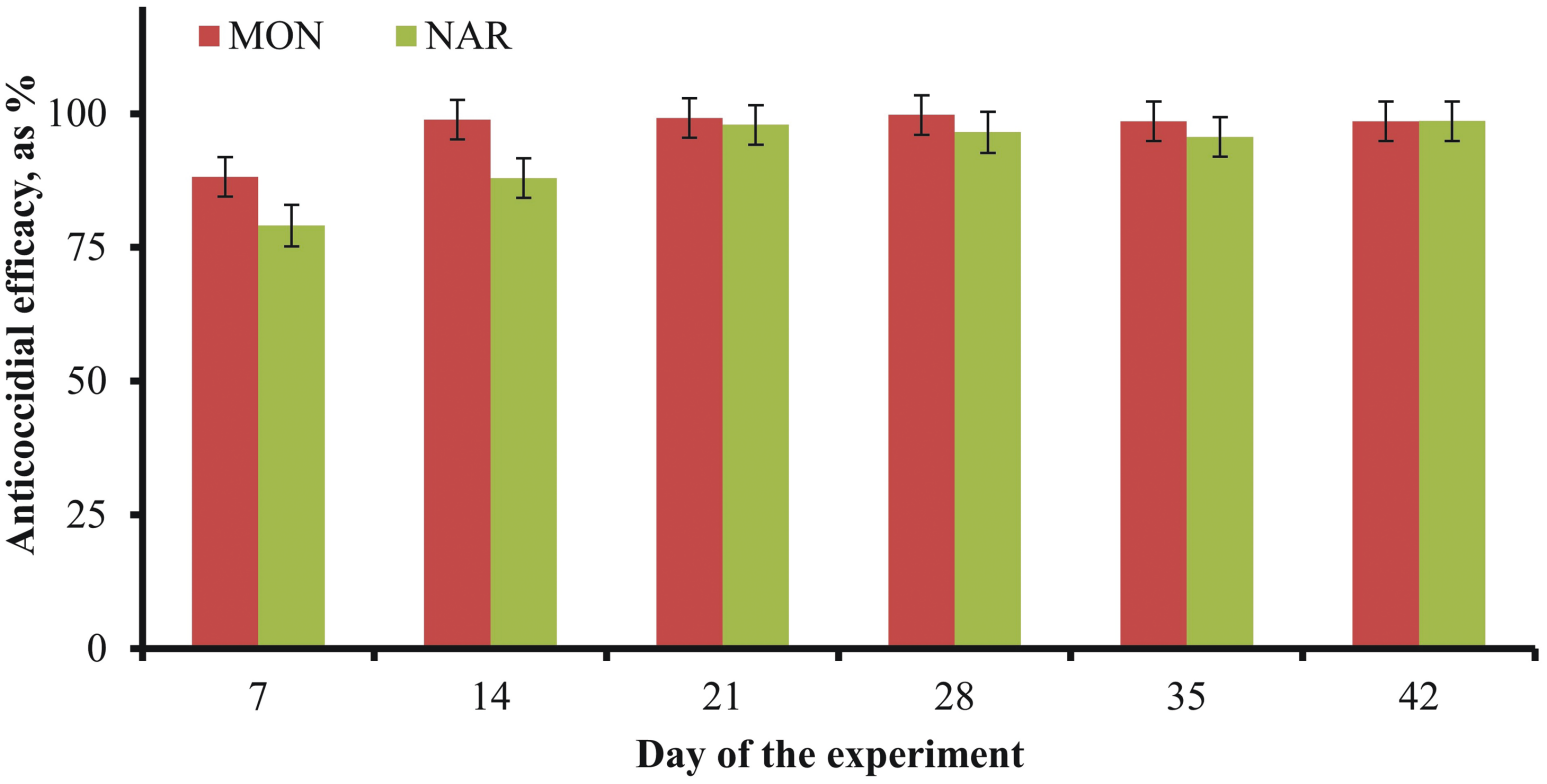
Anticoccidial efficacy (as %) of narasin (**NAR**; *n* = 8) or monensin (**MON**; *n* = 8) to *Eimeria spp.* in naturally infected calves. Treatments were individually offered to calves from days 0 to 42 of the experiment via a limit-fed (200 g/head daily) grain-based supplement. Fecal samples were collected from each calf on consecutive days (days −2 and −1, 6 and 7, 13 and 14, 20 and 21, 27 and 28, 34 and 35, and 41 and 42) and analyzed for oocysts per gram of feces (**OPG**; Ueno and Gonçalves, 1988). Values of OPG from samples collected in consecutive days were averaged, and referred as days −1, 7, 14, 21, 28, 35, and 42 to facilitate description of results. Anticoccidial efficacy was determined according to OPG results of calves within collection day, using results from non-supplemented calves (**CON**) as reference and the following equation: Efficacy = (OPG of CON—OPG of NAR or MON)/ OPG of CON (Lopes et al., 2014). No treatment effects nor treatment × day interaction were detected (*P ≥ *0.16).

The prevalence of individual *Eimeria* spp. in pooled fecal samples is described in Table 2, and was used to calculate anticoccidial efficacy according to each pathogenic species. In general, CON calves remained primarily infected by *E. bovis* and *E. zuernii* during the experiment, whereas the profile of *Eimeria* spp. infection changed in NAR and MON calves as prevalence of *E. bovis* decreased. The anticoccidial efficacy of NAR and MON did not differ (*P *≥ 0.16) when calculated across all *Eimeria spp.* (Figure 2), or according to prevalence of *E. bovis* and *E. alabamensins* (Table 3). No treatment × day interactions were detected for these variables (*P *≥ 0.18), and the anticoccidial efficacy to *Eimeria* spp. is reported in Figure 2 to characterize the day effects (*P *< 0.01). A treatment × day interaction was detected (*P *= 0.04) for anticoccidial efficacy according to *E. alabamensis* prevalence, which was greater (*P *< 0.01) in MON calves on days 7 and 14, but did not differ (*P *≥ 0.40) between treatments during the remainder of the experiment. Collectively, treatment effects on anticoccidial efficacy corroborate those noted for fecal OPG analyses. Both NAR and MON had similar efficacy against all pathogenic *Eimeria* spp. upon a 42-d feeding period, although MON was more effective in controlling *E*. *zuernii* during the initial 14 d of treatment. Perhaps the reason why MON calves had less OPG compared with NAR cohorts early in the experiment is related, at least partially, to hastened anticoccidial efficacy of monensin against *E*. *zuernii*.

**Table 3.** Anticoccidial efficacy (as %) of narasin (**NAR**; *n* = 8) or monensin (**MON**; *n* = 8) according to pathogenic *Eimeria* spp. in naturally infected calves.^*^^,^^†^^,^^‡^

Item	MON	NAR	SEM	*P-value*
*E. bovis*	98.4	94.5	1.8	0.15
*E. zuernii*				
day 7	95.8	85.7	2.1	<0.01
day 14	99.5	91.7	2.1	0.01
day 21	99.8	99.1	2.1	0.81
day 28	100.0	97.5	2.1	0.40
day 35	100.0	100.0	2.1	1.00
day 42	97.2	98.1	2.1	0.77
*E. alabamensis*	88.8	70.7	9.2	0.18

^*^Treatments were individually offered to calves from days 0 to 42 of the experiment via a limit-fed (200 g/head daily) grain-based supplement. Narasin and monensin were offered at, respectively, 0.8 mg/kg of BW (6.7 mg of Zimprova^TM^/kg of BW daily; Elanco Saúde Animal; São Paulo, Brazil) and 1 mg/kg of BW (5 mg of Rumensin^TM^/kg of BW daily; Elanco Saúde Animal).

^†^Fecal samples were collected from each calf on consecutive days (days −2 and −1, 6 and 7, 13 and 14, 20 and 21, 27 and 28, 34 and 35, and 41 and 42) and analyzed for oocysts per gram of feces (**OPG**; Ueno and Gonçalves, 1988). Values of OPG from samples collected in consecutive days were averaged, and referred as days −1, 7, 14, 21, 28, 35, and 42 (as BW assessment) to facilitate description of results. Anticoccidial efficacy was determined according to OPG results of calves within collection day, using results from non-supplemented calves (**CON**) as reference, and the following equation: Efficacy (as %) = (OPG of CON—OPG of NAR or MON)/ OPG of CON (Lopes et al., 2014).

^‡^A treatment × day interaction was detected (*P *= 0.04) for anticoccidial efficacy against *E. zuernii*, but not (*P ≥ *0.18) for *E. bovis* and *E. alabamensis*.

No treatment effects were detected (*P *≥ 0.51) for calf BW or growth rate during the experiment (Table 1), despite the known benefits of monensin and narasin to BW gain in cattle (Marques and Cooke, 2021) and the negative consequences of coccidiosis to cattle growth (Fitzgerald, 1980; Cornelissen et al., 1995; Oliveira et al., 2021). This experiment, however, was mainly designed to evaluate anticoccidial effects rather than performance responses. In a field study conducted concurrently with this experiment (Supplementary Table 1), *B. indicus*-influenced beef calves received supplementation with or without narasin inclusion for 150 d via creep-feeding (70 d of age until weaning). Narasin supplementation reduced OPG values by an average of 50% during the field study, with an overall anticoccidial efficacy of 82%. Narasin supplementation also increased calf growth rate by 10% and weaning BW by 6%, and these improvements are equivalent to results from research studies that evaluated narasin in growing beef cattle (Limede et al., 2021; Miszura et al., 2023).

Collectively, this experiment provides novel information regarding the use of narasin to control coccidiosis in naturally infected calves. All calves initiated the experiment with OPG levels above the threshold used to recognize coccidiosis as a health concern in the herd. Monensin was used as positive control, given its well-established anticoccidial properties in cattle. Both ionophores were similarly effective in controlling coccidiosis upon completion of the 42-d study, although the anticoccidial effects of monensin were noted earlier in the experiment. Nonetheless, results from this experiment demonstrate that narasin is an effective anticoccidial ionophore for naturally infected calves.

## Supplementary Material

txae069_suppl_Supplementary_Table
