# Supplementary material for: Supplementing narasin or monensin to control coccidiosis in naturally infected calves
Source: Transl Anim Sci. 2024 Apr 26;8:txae069. doi: 10.1093/tas/txae069 (PMC11127485; doi:10.1093/tas/txae069)
Supplement: txae069_suppl_Supplementary_Table [file txae069_suppl_supplementary_table.docx]

**Supplementary Table 1.** Performance responses and oocyst count per gram of feces (**OPG;** *Eimeria* spp.) in naturally infected calves receiving narasin (**NAR**) or no ionophore (**CON**).^1^

| **Item** | **CON** | **NAR** |
| --- | --- | --- |
| Number of calves | 29 | 29 |
| Age |  |  |
| d 0 (initial), d | 71.0 ± 1.7 | 72.6 ± 1.5 |
| d 150 (weaning), d | 221 ± 1.5 | 222 ± 1.4 |
|  |  |  |
| BW^2^ |  |  |
| d 0 (initial), kg | 89.7 ± 4.2 | 90.8 ± 2.8 |
| d 30, kg | 107 ± 3.9 | 115 ± 4.8 |
| d 60, kg | 134 ± 4.9 | 142 ± 5.5 |
| d 90, kg | 162 ± 5.2 | 171 ± 5.6 |
| d 120, kg | 191 ± 6.8 | 202 ± 5.9 |
| d 150 (weaning), kg | 211 ± 7.5 | 224 ± 7.8 |
| Growth rate, kg/d | 0.832 ± 0.047 | 0.914 ± 0.042 |
|  |  |  |
| OPG,^3^ log10 basis |  |  |
| d 0 (initial) | 1.02 ± 0.28 | 0.972 ± 0.242 |
| d 30 | 1.81 ± 0.27 | 0.936 ± 0.200 |
| d 60 | 1.64 ± 0.24 | 0.502 ± 0.170 |
| d 90 | 1.57 ± 0.25 | 1.00 ± 0.21 |
| d 120 | 1.58 ± 0.25 | 1.07 ± 0.21 |
| d 150 (weaning) | 1.23 ± 0.21 | 0.376 ± 0.157 |
|  |  |  |

^1^ Calves were maintained with their dams in 2 separate pastures according to treatment, and rotated pastures every 30 d. Treatments were offered to calves via creep-feeding from d 0 to 150 of the experiment. Narasin was added to the creep-feeding supplement at 13 mg/kg of dry matter (108 mg of Zimprova^TM^/kg of dry matter; Elanco Saúde Animal; São Paulo, Brazil). Values reported are means ± SEM

^2^ Body weight (**BW**) was unshrunk, and individual growth rate was modeled by linear regression of BW against sampling days (d 0 to d 150).

^3^ Fecal samples were analyzed for *Eimeria spp.* OPG (Ueno and Gonçalves, 1998). The OPG (observed egg count + 1) of each calf was log-transformed before statistical analyses and reported as log10 (Oliveira et al., 2020).
